# Comparative Production Analysis of Three Phlebovirus Nucleoproteins under Denaturing or Non-Denaturing Conditions for Crystallographic Studies

**DOI:** 10.1371/journal.pntd.0000936

**Published:** 2011-01-04

**Authors:** Violaine Lantez, Karen Dalle, Rémi Charrel, Cécile Baronti, Bruno Canard, Bruno Coutard

**Affiliations:** 1 Architecture et Fonction des Macromolécules Biologiques, UMR 6098 Centre National de la Recherche Scientifique, Université de la Méditerranée and Université de Provence, Marseille, France; 2 Unité des Virus Emergents, UMR 190, Aix-Marseille Université and Institut de Recherche pour le Développement, Marseille, France; Tulane School of Public Health and Tropical Medicine, United States of America

## Abstract

Nucleoproteins (NPs) encapsidate the Phlebovirus genomic (-)RNA. Upon recombinant expression, NPs tend to form heterogeneous oligomers impeding characterization of the encapsidation process through crystallographic studies. To overcome this problem, we set up a standard protocol in which production under both non-denaturing and denaturing/refolding conditions can be investigated and compared. The protocol was applied for three phlebovirus NPs, allowing an optimized production strategy for each of them. Remarkably, the Rift Valley fever virus NP was purified as a trimer under native conditions and yielded protein crystals whereas the refolded version could be purified as a dimer. Yields of trimeric Toscana virus NP were higher from denaturing than from native condition and lead to crystals. The production of Sandfly Fever Sicilian virus NP failed in both protocols. The comparative protocols described here should help in rationally choosing between denaturing or non-denaturing conditions, which would finally result in the most appropriate and relevant oligomerized protein species. The structure of the Rift Valley fever virus NP has been recently published using a refolded monomeric protein and we believe that the process we devised will contribute to shed light in the genome encapsidation process, a key stage in the viral life cycle.

## Author Summary

Phleboviruses have a worldwide distribution and are usually represented by their prototype Rift Valley fever virus that can have a great impact on health and economy in Africa. The genome of phleboviruses is a segmented negative strand RNA that is encapsidated by the nucleoprotein. The structure of the monomeric nucleoprotein has been recently published but it's not sufficient to decipher a convincing mechanism for the nucleoprotein oligomerization. In order to understand this key step in the virus life cycle, the purification of oligomers homogeneous in size would be a key step to launch structural studies. To that aim, a procedure relying on recombinant protein production in both denaturing and non-denaturing conditions was applied to three phlebovirus nucleoproteins. Although the best production pipeline differs for each protein, pure and homogeneous solutions of Rift Valley fever virus and Toscana virus nucleoproteins were successfully obtained. Both proteins, behaving as apparent trimers in solution, lead to protein crystallization, a starting point to understand the genome encapsidation through structural studies.

## Introduction

**Figure 1:**
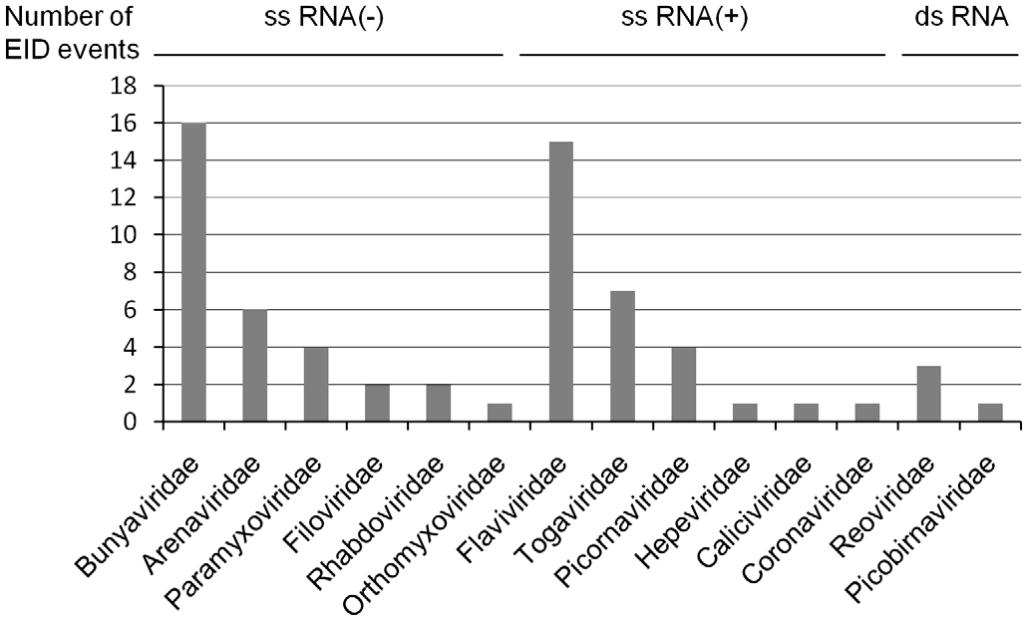
Number of emerging infectious diseases (EIDs) related to RNA viruses (1940–2004) period [**11**]. Virus families are organised by genome structure: negative single strand RNA (ss RNA(-)) positive single strand RNA (ss RNA(+)), and double strand RNA (ds RNA).

The genus *Phlebovirus* belongs to the *Bunyaviridae* family that includes four others genera, namely *Hantavirus*, *Nairovirus*, *Orthobunyavirus*, and *Tospovirus*. Phleboviruses have a worldwide distribution and are associated with a wide variety of arthropods (sandflies, mosquitoes, ticks). Rift Valley fever virus (RVFV), the prototype species of the genus, is endemic in Africa, where it is zoonotic, infecting mainly sheep, and causing severe disease with high rates death through abortion in sheep. During these outbreaks, RVFV can pass to human either directly via abortion products or via mosquito transmission, leading occasionally to potentially fatal meningoencephalitis and/or haemorrhagic fevers. Recent outbreaks occurred in the horn of Africa [1]–[2] and the virus also spread into the Arabic peninsula [3]. Outbreaks are directly correlated to rainfalls in these regions and thus, climate and vegetation data may be used to predict areas and periods at risk [4]. In the Mediterranean basin phleboviruses other than RVFV are well established, and seroprevalence can in some regions reach 20% in man [5]. Phleboviruses are mainly represented by Sandfly Fever Sicilian virus (SFSV), Sandfly Fever Naples virus (SFNV), Toscana virus (TOSV) and viruses more or less distantly related to SFNV and SFSV [6], [7], [8]. These phleboviruses are transmitted by phlebotomine flies in regions where the latter circulate. SFN- and SFS-like viruses can cause mild febrile illnesses (sometimes paucisymptomatic) which are likely to be largely underestimated due to the lack of diagnosis, little awareness among health professionals. Toscana virus is in the top three viral etiologies of aseptic meningitis in Italy, Spain and France [9]–[10]. In this respect, phleboviruses are representative of *Bunyaviridae*. Among the viral world, this family was the major responsible for emerging infectious diseases (EIDs) events between 1940 and 2004 (figure 1, data extracted from [11]), even more represented than the *Flaviviridae*. Therefore, efforts should be brought not only in diagnosis but also in the understanding of the viral cycle to emphasize antiviral research against these viruses.

Phleboviruses, and in general members of the family *Bunyaviridae*, are enveloped spherical viruses of icosahedral geometry [12]–[13] with an 80–120 nm long diameter. Their genome is made of three segments [14]. Among these segments, two are single negative stranded (M and L) whereas the third one (S) can adopt an ambisense strategy of expression [15]. The L fragment is encoding the L protein that is carrying the RNA dependent RNA polymerase activity involved in the primary and secondary transcription generating mRNA and replicative intermediates, respectively. In complement, L is also presumably carrying the cap snatching activity required in the viral mRNA capping [16]. The M segment is coding for the glycoprotein precursor that is cleaved by host proteases in two structural domains G_C_ and G_N_ [17]. The S fragment can code for the non structural protein NSs as well as the nucleoprotein NP protein. The nucleoprotein is a 245 amino acids protein (for Rift Valley fever virus) that can bind to genomic RNA and replicative intermediates to form ribonucleoproteic complexes (RNP) of circular appearance [18]–[19]. The NP oligomerization and its RNA binding properties have not been extensively described until recently. NPs can dimerize with the involvement of their N-terminal domain and this NP-NP interaction does not depend on the presence of RNA [20]–[21]. To date, no physiological multimeric stage over dimerization has been clearly identified. Biophysical and structural studies of the NP alone would therefore provide insights into RNP formation process. For such studies, it would be beneficial to purify homogeneous preparations of monomeric nucleoprotein, or at least, NP assemblies of tractable, defined multimerization status.

Many previous studies reported the expression and purification of nucleoproteins for various applications. The NP protein of RVFV can be expressed in insect cells using the recombinant baculovirus technology, but the protein forms a high molecular weight RNP complex, as shown by size exclusion chromatography [22]. The NP proteins have already been produced in *E. coli* for ELISA experiments. The proteins were purified under denaturing conditions [23], with a large N-terminal non cleavable tag [24], or the purification procedure was stopped after the first affinity purification step [25]. Only recently, RVFV NP was purified by refolding the recombinant protein while the natively produced protein was considered as heterogeneous. The refolded protein lead to the first crystal structure determination of the phlebovirus NP [26]. In solution, the refolded protein behaved as a monomer and the NP crystallized as a dimer that was thought to occur naturally. Nevertheless, in this study, the role and the presence of the dimer in the NP oligomeric form observed by electron microscopy remained unclear.

In order to further understand the structural properties of phlebovirus NP, it is necessary to set up a process that would lead to the production of protein oligomers pure and homogeneous in size, as previously performed for the rabies NP [27]. Additionally, the process would include a tag removal to improve crystallization. To that aim, and based on the existing results, we decided to evaluate two strategies relying on bacterial recombinant expression for the production of several phlebovirus NP protein suitable for structural studies. These strategies already met success in large scale structural genomics projects. Firstly, the screening of N-terminal tags can drastically improve soluble expression [28]–[29]. Secondly, when proteins are reluctant to soluble expression, they can be expressed as inclusion bodies (IB) before being refolded in non-denaturing conditions [30]–[31]. In this study, both strategies will be performed in parallel, even if proteins can be expressed in the soluble fraction, in order to provide comparative data suitable to design optimized production protocols for NP proteins. In addition to the production of NPs suitable for structural studies, these data may highlight trends in the larger field of recombinant protein expression.

## Materials and Methods

### Cloning and protein expression

**Figure 2:**
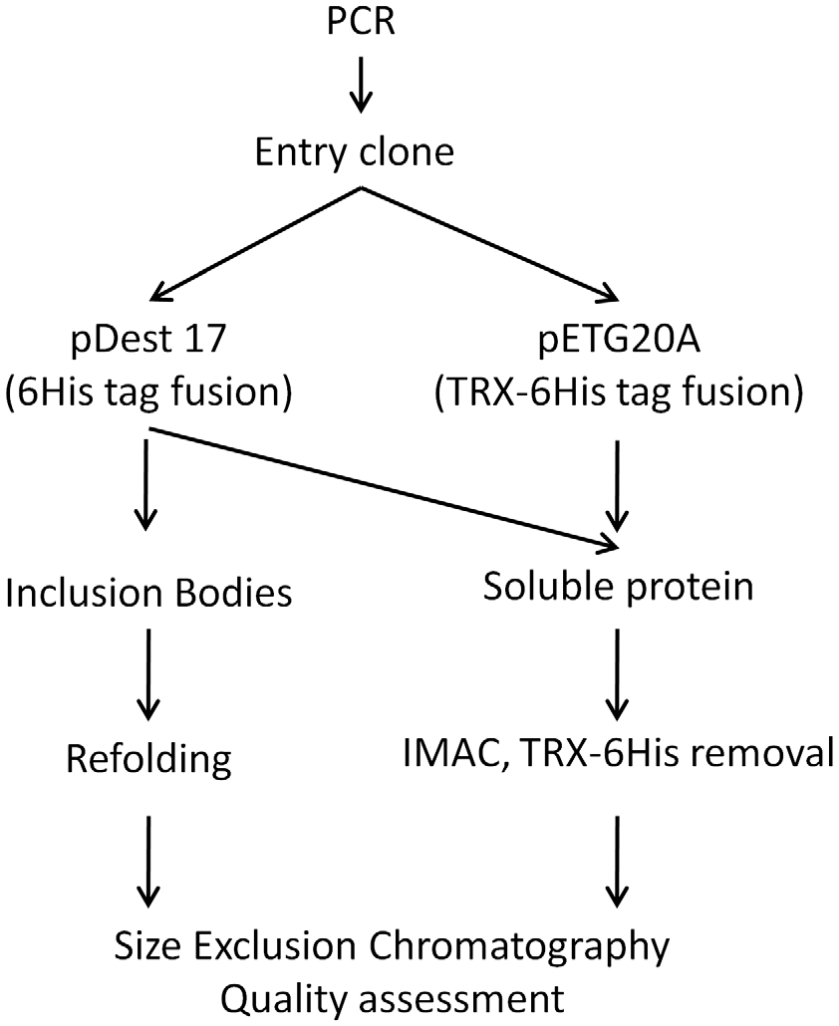
Production process with in-parallel denaturing and non-denaturing procedures applied to the three nucleoproteins.

cDNA corresponding to the three Nucleoproteins (NP) of Rift Valley fever virus (RVFV, strain Smithburn DQ380157.1), Sandfly Fever Sicilian virus (SFSV, strain J04418.1) and Toscana virus (TOSV, strain AR2005) were amplified using two Polymerase Chain Reactions (PCR). A first amplification was performed using i) a forward primer carrying the coding sequence of the Tobacco Etch Virus (TEV) protease cleavage site followed by the 21 nucleotides of the NP sequences (5′ GAAAACCTGTACTTCCAGGGT-21 nt 3′) and ii) a reverse primer carrying attB2 sequence for cloning by recombination, two stop codons and the 21 nucleotides long reverse complement sequence of the NP (5′ GGGGACCACTTTGTACAAGAAAGCTGGGTC TTATTA -21 nt reverse complement 3′). A second PCR was done on the first PCR product using the same reverse primer and a universal forward primer carrying the attB1 sequence as well as two additional nucleotides (TA) for the coding frame, and a sequence hybridizing the TEV protease cleavage site (5′ GGGGACAAGTTTGTACAAAAAAGCAGGCT TA GAAAACCTGTACTTCCAGGGT 3′). This second PCR products were cloned into the pDonR201 plasmid by recombination (Gateway, Invitrogen). The resulting entry clones were sequenced. The entry clones were then used as templates to clone the NP into two expression plasmids (see figure 2): pDest17 (Invitrogen), that allows the expression of the NP in fusion with a N-terminal Hexahistidine (6His) tag, removable with the inserted TEV protease cleavage site and pETG20A (kindly provided by Dr A. Geerlof) that allows the expression of the NP in fusion with a removable Thioredoxin-Hexahistidine (TRX-6His) tag. The resulting expression plasmids were transformed in C41 (DE3) *E. coli* strain (Avidis SA) carrying the pRARE plasmid (Novagen). For each construct, one liter of Terrific Broth (Athena Enzymes) containing 100 mg/l of ampicillin and 34 mg/l of chloramphenicol was inoculated with 30 ml of an overnight pre-culture. The bacteria were grown at 37°C up to OD_600 nm_ reached 0.8. Recombinant protein expression was then induced by adding 0.5 mM isopropyl β-D-1-thiogalactopyranoside (IPTG) and the culture temperature was dropped to 17°C for 16 hours. Cells were harvested by centrifugation at 4 000 g, 10 minutes. Cell pellets were then resuspended in 50 mM Tris buffer, 300 mM NaCl, 10 mM imidazole, 0.1% Triton, and 5% glycerol (pH 8.0). Lysozyme (0.25 mg/ml), phenylmethylsulfonyl fluoride (1 mM), DNase I (2 µg/ml), and EDTA-free protease cocktail (Roche) were added before performing a sonication step. The lysates were centrifuged at 12 000 g for 45 minutes. For both 6His and TRX-6His constructs, the supernatants were collected for the purification procedure in non-denaturing conditions, whereas for the 6His constructs, the pellets were used for the purification process in denaturing conditions, as described in figure 2.

### Purification under non-denaturing conditions

The recombinant soluble proteins were purified from the supernatant previously recovered using the Akta Xpress fast purification liquid chromatography system (GE Healthcare) as follows. The first purification step (immobilized metal affinity chromatography) was performed on a 5-ml His prep column (GE Healthcare). The clarified bacterial lysates were loaded at 5 ml/min. The columns were then washed with a washing buffer (50 mM Tris, 300 mM NaCl and 50 mM imidazole pH 8.0) and the proteins were eluted with 50 mM Tris, 300 mM NaCl and 500 mM imidazole pH 8.0. The elution fraction (15 ml) was then dialyzed against a buffer compatible with the TEV protease activity (Hepes 10 mM, NaCl 300 mM, pH 7.5). 6His tagged TEV protease mutant selected for optimized expression [32] was added to the protein samples in a 1/20 (w/w) ratio and cleavage was performed 16 hours à 4°C. The tag removal efficacy was evaluated on a Coomassie blue stained SDS Page gel by ImageJ software and was calculated on the band surface and intensity of the full lengths TRX-6His fusions: [100-(I_AC_S_AC_/I_BC_S_BC_)x100]% where I is the intensity of the band, S is the surface, “AC” is “After Cleavage” and “BC” is “Before Cleavage”. After cleavage, the solution was loaded on a 5-ml His prep column and the cleaved proteins were collected in the flow through, whereas 6His-TEV protease, TRX-6His tag and not cleaved fusions were retained onto the column. The cleaved proteins were further purified by a Size Exclusion Chromatography (SEC) in 10 mM Hepes, 300 mM NaCl, pH 7.5 using a 16/60 Superdex 75 (GE Healthcare). The SEC was calibrated using the LMW and HMW calibration kits (GE Healthcare) in order to convert elution volumes in Molecular Weights. The purity of the samples was checked on Coomassie blue stained SDS Page gels. The fraction of the low molecular weight oligomer of NP (LMWNP) amenable to protein crystallization was defined as: (PA_LMWNP_/PA_ALL_x100)% where PA is the “peak area” of the SEC chromatogram at OD_280 nm_. The identity of the recombinant proteins were confirmed by matrix-assisted laser desorption ionization-time of flight mass (MALDI-TOF, Bruker Autoflex, Bruker Daltonics,) spectrometry after trypsin digestion. Finally, the oligomerization was also assessed on Coomassie blue stained SDS Page gels, with an upstream treatment with glutaraldehyde, as previously described [20]. Briefly, different collected fractions were incubated 30 minutes at room temperature with glutaraldehyde at a 0.05% concentration. The samples were then denatured 5 minutes at 95°C in SDS Page sample buffer (100 mM Tris pH 6.8, 25% glycerol, 10% sodium dodecyl sulfate, 5 mM β-mercaptoethanol, bromophenol blue) prior electrophoresis on SDS Page gels.

### Purification under denaturing conditions

The pellets obtained from the centrifuged cell lysates were washed twice to purify the inclusion bodies. The pellets were resuspended in a first washing buffer (50 mM Tris, 25 mM Imidazole, 300 mM NaCl, 1 M urea, 0.1% Triton, pH 8) and centrifuged at 12 000 g during 30 minutes. The supernatant was removed and the new pellets were resuspended in a second washing buffer (50 mM Tris, 25 mM Imidazole, 300 mM NaCl, 1 M urea, pH 8). After a second centrifugation step (12 000 g, 30 minutes), inclusion bodies recovered from the pellets were solubilized in a denaturing buffer (50 mM Tris, 300 mM NaCl, 25 mM Imidazole, 8 M Guanidium, pH 8). The purity of the recombinant proteins was assessed on Coomassie blue stained SDS Page gels and their quantity was evaluated at OD_280 nm_. Denaturing proteins were concentrated up to about 10 mg/ml and diluted 1/20 (v/v) in a refolding buffer (50 mM Sodium acetate, 100 mM KCl, 10 mM β-mercaptoethanol, pH 4.5) at 4°C overnight. The refolding volume did not exceed 60 ml (30 mg of the protein) in order to have protein quantities and volumes compatible with the downstream procedure. The observed aggregates after refolding were removed by centrifugation (10 000 g, 15 minutes) and the solution was further clarified using filtration on 0.22 µm filters. A preliminary refolding efficacy was calculated as follow: (100×[NP]_Sol_/[NP]_BR_)%, where “[NP]_Sol_” is the NP concentration in the soluble fraction after refolding and [NP]_BR_ is the NP concentration before refolding (0.5 mg/ml). The refolded proteins were then concentrated on Amicon Ultra 10 K (Milipore) up to 5 ml before being loaded on a 16/60 Superdex 75 (GE Healthcare) equilibrated with 10 mM Hepes, 300 mM NaCl, pH 7.5 for SEC purification and homogeneity analysis, as described previously.

### Protein crystallization

Crystallization trials were initiated with the RVFV NP purified under non-denaturing conditions and TOSV NP purified from IB using a nano-drop dispenser (Honeybee; Genomic Solutions) in 96-well sitting drop plates (Greiner Bio One). Three commercial crystallization kits were tested at 20°C: Structure Screen combination, Stura footprints (Molecular Dimensions Limited), and Nextal SM1 (Qiagen). For each condition, three drops were done: 300, 200 or 100 nl were added to 100 nl of the crystallization solution.

## Results

### Cloning and expression strategy

The ORFs encoding NP proteins of RVFV, SFSV and TOSV were cloned in pDONR201 before being re-introduced in two plasmids for expression as a N-terminal tag fusion, as described in figure 2. Based on their small size and ability to be purified by Immobilized Metal Affinity Chromatography (IMAC), only 6His (3.3 kDa) and TRX-6His (14.6 kDa) tags were selected for the tag screening although other tags such as 6His/MBP (Maltose Binding Protein), or 6His/GST (Glutathion S-Transferase) could be available and compatible with the cloning procedure[33]–[34]. Nevertheless, the latter tags were not tested because they are much larger than 6His or TRX-6His and might interfere in the oligomerization process by steric hindrance. When fused to a removable 6His tag, the NP proteins can follow two procedures. If the protein is expressed in the soluble fraction, it can be purified under non-denaturing conditions. The 6His tagged fusions can also be expressed as inclusion bodies for subsequent refolding. By contrast, the TRX-6His was used only for soluble expression for two reasons. Firstly, the TRX tag is expected to improve protein solubility. Secondly, if the TRX-6His tag needs to be refolded in a condition that isn't compatible with recombinant protein optimum, and *vice versa*, the presence of the tag would be deleterious for refolding efficacy. Protein expression was performed in only one culture condition that was chosen on the conclusions of a previous report [35]. Briefly, the two main culture parameters having an impact on soluble expression are the use of rare tRNA co-expressing strains to improve expression yields, and post-induction cultures at low temperatures to promote solubility. *E. coli* strain carrying a pRARE plasmid (Novagen) and bacterial growth at 17°C were thus selected.

### Protein purification under non-denaturing conditions

**Figure 3:**
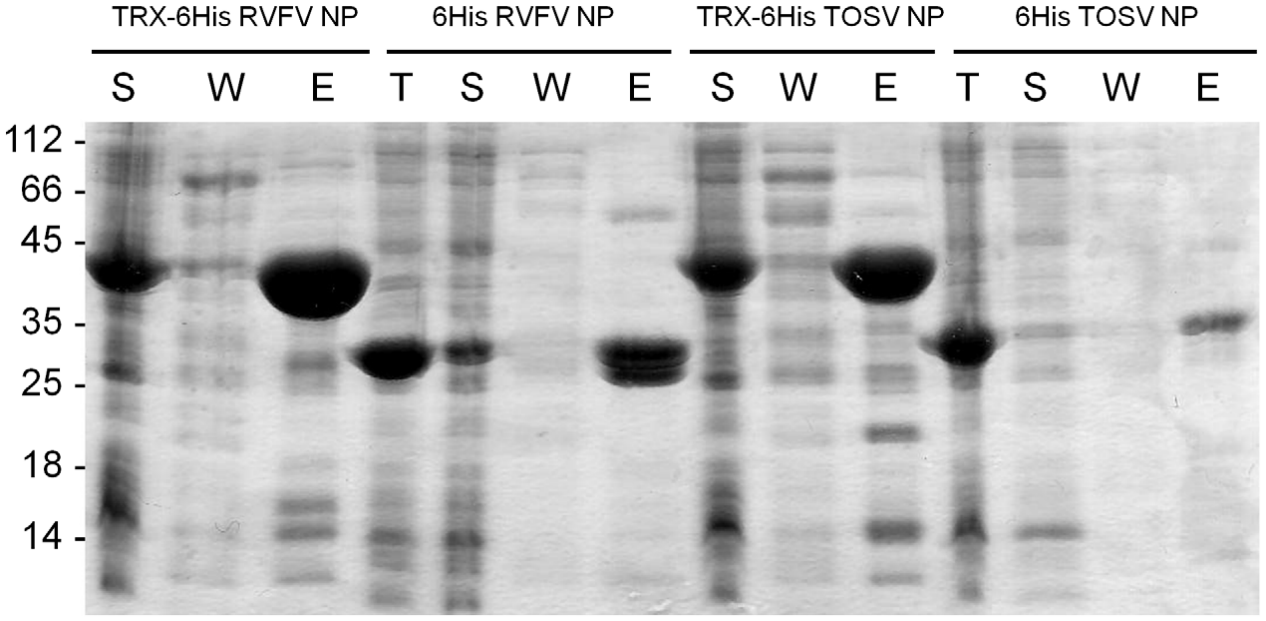
Expression levels and IMAC purification results of tagged RVFV and TOSV NPs. Analysis on a Coomassie blue stained SDS Page gel. S: Soluble fraction of the bacterial lysate; W: Wash step at 50 mM imidazole during the IMAC purification; E: Elution fraction of the IMAC; T: Total bacterial lysate. The calculated molecular weights of RVFV, SFSV and TOSV NPs without the tags are respectively 27.4, 27.9 and 27.7 kDa. The calculated molecular weights of the cleavable 6His and TRX-6His tags are respectively 3.3 and 14.6 kDa.

For each NP fused with an Nterminus 6His tag, a part of the recombinant protein can be expressed in its soluble form, as shown by the amount of protein quantified from the soluble fraction after IMAC purification (figure 3, data summarized in table1). Among the 3 NPs, RVFV NP was the only one to be soluble at yields above 1 mg/L culture. This latter yield was arbitrarily defined as the threshold compatible with downstream crystallogenesis experiments. Therefore, among the 6His tagged fused proteins, only RVFV NP was further purified and characterized. When compared to the amount of the insoluble fraction (data in table 1), the soluble fraction of NPs corresponds to 1.5% of the overall expression (3.5 mg in the soluble fraction compared to 240 mg in the inclusion bodies for RVFV NP), or less for SFSV and TOSV NPs. By contrast, when fused to the solubilizing TRX-6His tag, the NPs are 7 to 20-fold more soluble, providing thus enough material for tag cleavage. Interestingly, the solubility trend observed with the 6His fusions (RVFV>TOSV>SFSV) is conserved with the TRX-6His tag.

**Table 1:** Production results of the Nucleoproteins (NPs) of Rift Valley fever virus (RVFV), Sandly Fever Sicilian virus (SFSV) and Toscana virus (TOSV).

		RVFV NP		SFSV NP		TosV NP	
		6His Tag	TRX-6His	6His Tag	TRX-6His	6His Tag	TRX-6His
Native conditions	Soluble expression	3.5 mg	26 mg*	<0,5 mg	5 mg*	1 mg	20 mg*
	Tag removal	N.A	87%	N.A	<10%	N.A	100%
	LMW NP vs all NP	46%	33%	N.A	N.A	N.A	5%
	Final quantity of LMW NP	**1.4 mg**	**6 mg**	N.A	N.A	N.A	**0.9 mg**
	LMW oligomer	**Trimer**	**Trimer**	N.A	N.A	N.A	**Trimer**
Denaturing conditions	Insoluble expression	240 mg		209 mg		234 mg	
	IB refolding	29%		0%		26%	
	LMW NP vs all NP	16%		N.A		63%	
	Final quantity of LMW NP	**0.7 mg**		N.A		**4 mg**	
	LMW oligomer	**Dimer**		N.A		**Trimer**	

The result at key steps of the process was evaluated. N.A: not applicable, (*): quantities are indicated after the subtraction of the TRX contribution, so figures can be directly compared, irrespective to the nature of the tag.

**Figure 4:**
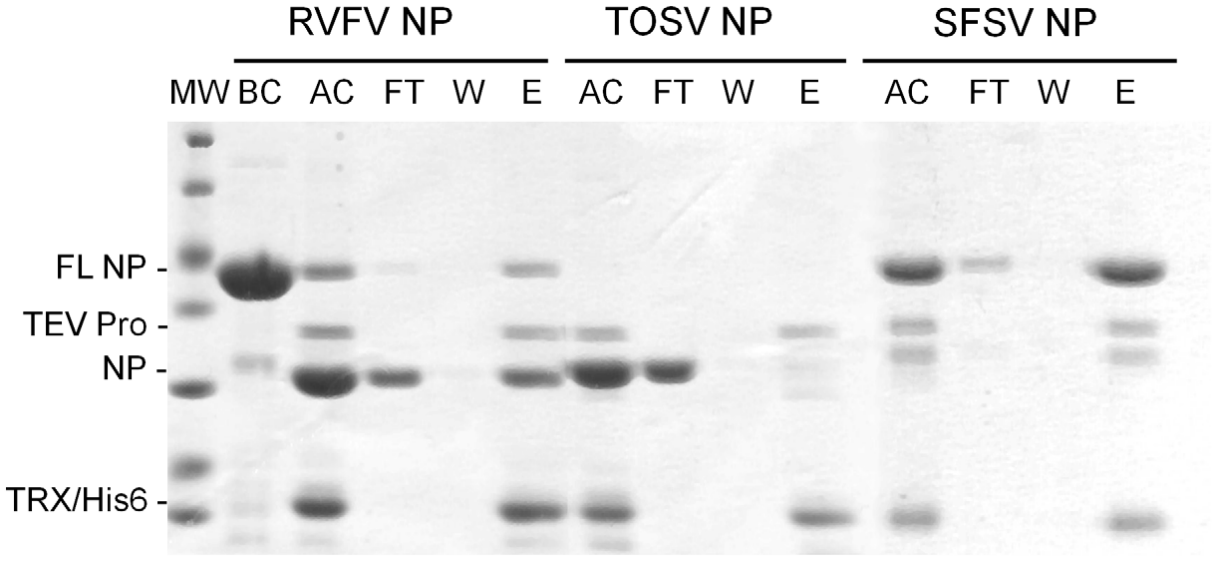
TRX-6His tag removal by TEV protease analysed on Coomassie blue stained SDS Page gel. BC: Before cleavage; AF: After cleavage; FT: Flow Through fraction of the IMAC; W: Wash step at 50 mM imidazole during the IMAC purification; E: Elution fraction of the IMAC; FL NP: Full length TRX-6His construct; TEV Pro: TEV protease; MW: Molecular weight marker, same ladder as figure 3.

The removal of the TRX-6His tag was then assessed based on two criteria. First, the remaining full length TRX-6His-NP can be quantified and compared to the quantities of the full length constructs before cleavage. Using this calculation, the best cleavage efficacy was observed for TOSV NP, for which no residual full length fusion protein was observed after the TEV protease cleavage, as shown in figure 4. RVFV NP was also efficiently recovered since only 13% of the full length was reluctant to TEV protease cleavage. In contrast with to these two NPs, more than 90% of the full length TRX-6His-SFSV NP remained uncleaved after the incubation with TEV protease, resulting in about 10% cleavage yield. Following the cleavage, the protein solution is loaded again on a Nickel immobilized column. Theoretically, this step binds 6His tagged proteins (TRX-6His, full length fusions, and TEV protease) and separates them from the untagged protein (cleaved protein of interest) going through the column. Practically, most of the cleaved TOSV NP was found in the flow through fraction whereas the 6His tagged proteins and a small amount of cleaved TOSV NP were trapped on the column and released during elution (figure 4). The purification of RVFV NP was not as efficient as for TOSV NP. Indeed, cross contaminations of full length and cleaved protein can be found in both flow through and elution pools. However, the cleaved RVFV NP represents about 90% of the flow through and is therefore amenable to SEC. For SFSV NP, the cleaved protein was observed at the elution of the IMAC with the TRX-6His-NP fusion. Moreover, only non cleaved NP went through the nickel immobilized column. It was thus concluded that the purification of SFSV NP failed with the TRX-6His construct and the process was aborted.

In summary, among the six fusion proteins tested from the soluble fraction, three constructs yielded amounts and homogeneity criteria, as shown in table 1: 6His-RVFV NP, TRX-6His-RVFV NP and TRX-6His-TOSV NP.

**Figure 5:**
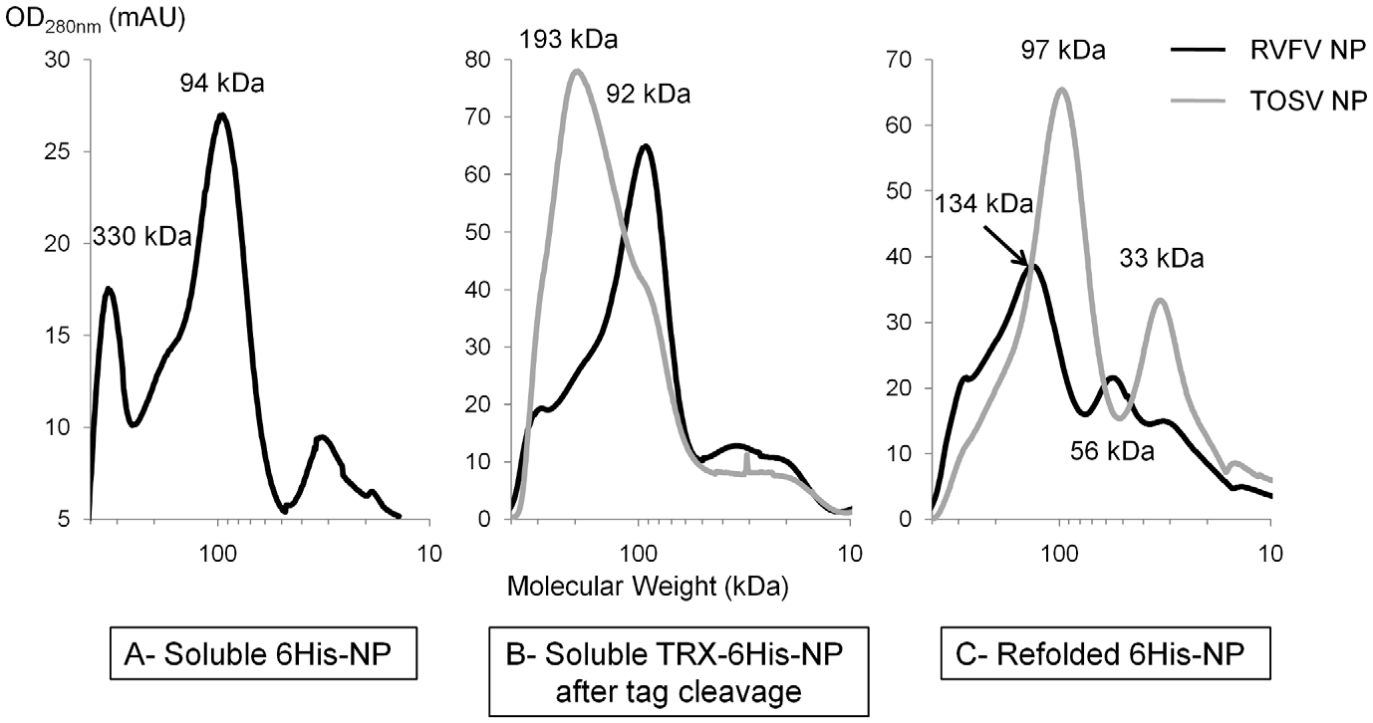
SEC chromatograms at OD_280 nm_ for RVFV and TOSV NPs produced in different conditions. The molecular weights have been extrapolated from the elution volumes using a calibration curve. Panel A corresponds to SEC chromatogram of 6His constructs purified in non-denaturing conditions, Panel B: TRX-6His constructs in non-denaturing conditions, Panel C: 6His constructs after refolding.

6His-RVFV NP showed a degradation pattern after the IMAC purification, resulting in four sub-products observed from 26 to 30 kDa (figure 3), whereas the expected size of the NP with the 6His Tag is 31 kDa. The oligomerization of the sub-products was then analyzed by SEC (figure 5, panel A). Based on the calibration curve, the main part of the 6His-RVFV NP eluted at 94 kDa that could correspond to a trimer of 6His-NP (theoretical MW: 93 kDa). A larger oligomer over 300 kDa was also observed. The oligomerization of the NP is independent to the protein degradation since the four cleaved products are almost equally distributed along the chromatogram (figure 5, panel A). Therefore, although the criteria in homogeneity and quantity were reached (see Table 1), crystallization trials were not launched because of protein degradation.

**Figure 6:**
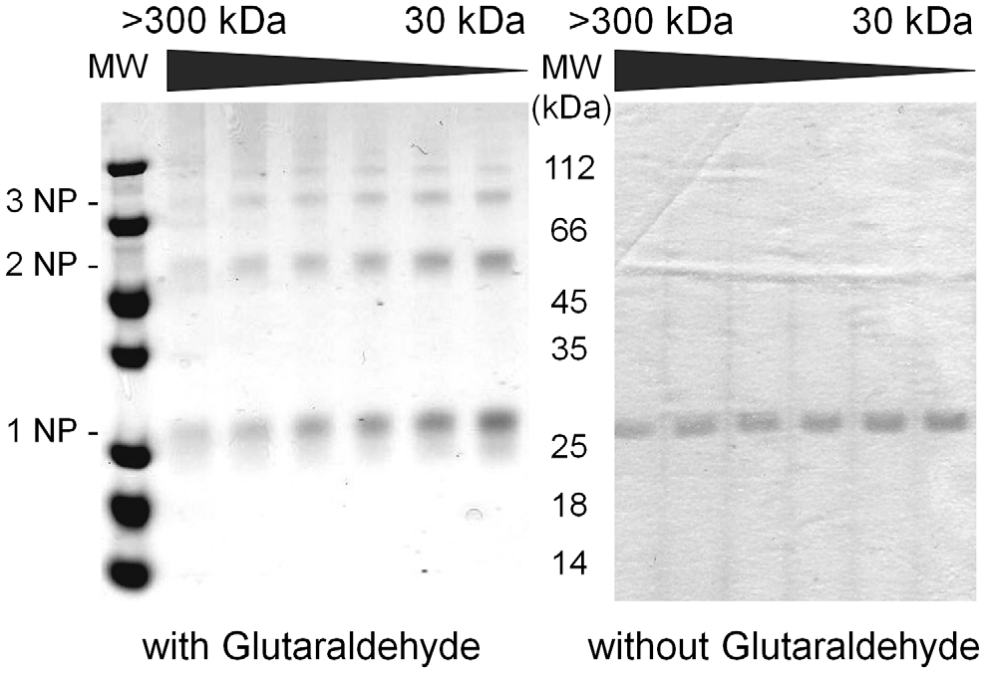
SEC fractions of RVFV NP with or without glutaraldehyde. Analysis on Coomassie blue stained SDS Page gel.

SEC was also performed for the NP of both RVFV and TOSV after release of the TRX-6His tag. RVFV NP mainly eluted at 93 kDa (figure 5, panel B) following the same trend as that of 6His-RVFV NP. In order to compare the oligomerization state of the RVFV NP in this protocol to a previous study [20], several fractions from 300 to 30 kDa were treated with or without glutaraldehyde and analyzed on Coomassie blue stained SDS Page (figure 6). The cross-linking with glutaraldehyde resulted in three major populations (monomers (1NP), dimers (2NP) and timers (3NP)) as well as to a lower extent tetramers and high molecular complexes on the top of the gel. The amounts of monomers, dimers, trimers and tetramers remained almost unchanged along the chromatogram. By contrast, crosslinked HMW complexes could be observed in the fractions that were collected at low elution volumes. Unlike the corresponding 6His tagged NP, the protein was pure and not degradated as judged by Coomassie Blue stained SDS Page (Figure 6). Fractions corresponding to the major peak were pooled and the so-called LMW NP gathered 33% of the injected NP. The behavior of TOSV NP after the TRX-6His tag cleavage was different: most of the protein eluted at 193 kDa and the LMW NP corresponds to about 5%, leading to less than 1 mg of protein (table 1). Nevertheless, the protein eluting at 193 kDa was pooled and concentrated for crystallogenesis experiments.

### Purification under denaturing conditions and refolding of IB

**Figure 7:**
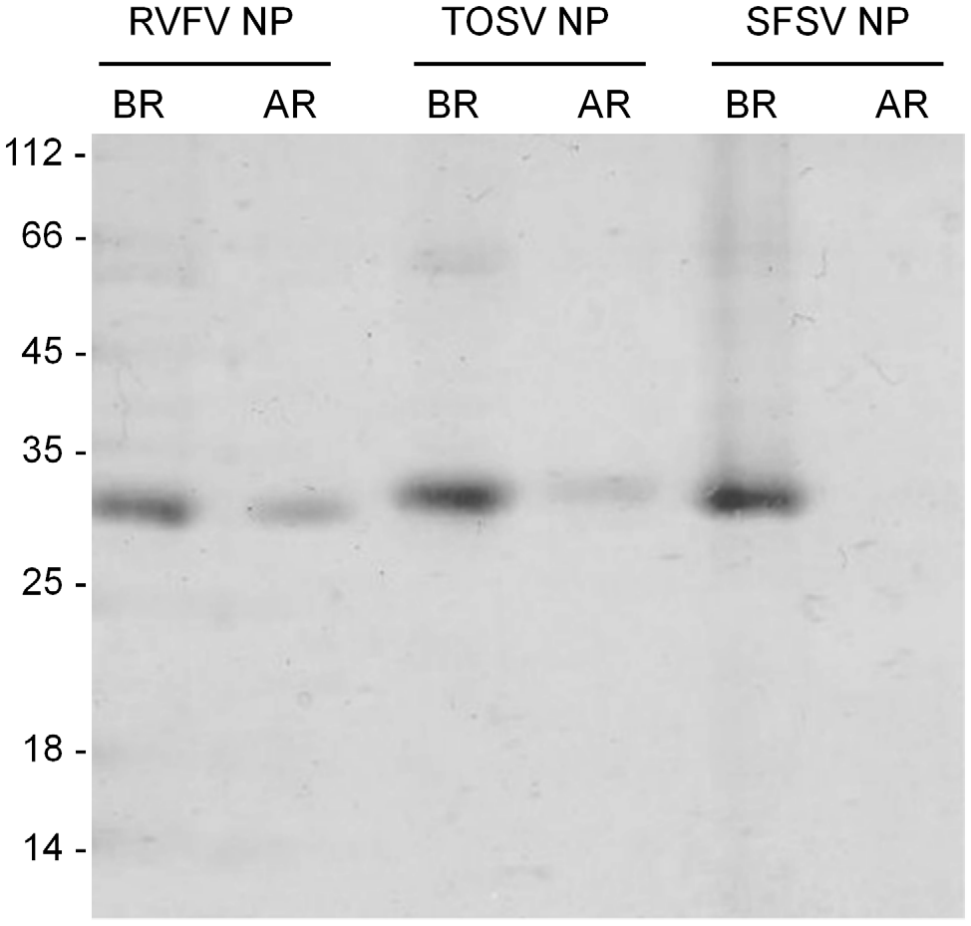
Refolding efficacy for the three NPs analysed on Coomassie blue stained SDS Page gel. BR: Before refolding; AR: soluble and filtrated fraction obtained after refolding.

More than 200 mg of each of the 6His-NP constructs were expressed as inclusions bodies (table 1). Since the recombinant proteins were highly expressed, two standard washes of the inclusion bodies were sufficient to recover NPs that are more than 90% pure, as shown in the total fraction of the refolded NPs (figure 7). Therefore, neither additional purification step nor optimized washes were needed before refolding. The theoretical isoelectric point (pI) for RVFV, SFSV and TOSV are respectively 9.8, 10.1 and 9.9. In order to refold 6His-NPs at a pH distant to the protein pI, it was decided to refold at a low pH and sodium acetate at pH 4.5 was selected. A first analysis of the protein refolding efficacy was performed by comparing the total amount of 6His-NP to be refolded (e.g. 30 mg for each NP) with the soluble and filtrated fraction after overnight dilution in the refolding buffer. Quantitative data (table 1) were obtained by comparing OD_280 nm_ in the total and soluble fractions. Qualitative data (protein purity, and degradation) were evaluated using Coomassie blue stained SDS Page (figure 7). More than 25% of RVFV and TOSV 6His-NP were recovered in the soluble fraction whereas the corresponding SFSV construct was completely insoluble after refolding.

The oligomerization states of RVFV 6His-NP after refolding was different to the ones observed for the soluble corresponding NPs. Most of the protein eluted at 134 kDa (4,3 molecules of 6His-NP) during the SEC. A minor peak at 56 kDa (1,8 6His-NP) was also observed (figure 5). This latter population corresponds to 16% of the injected protein, leading to less than 1 mg of protein from the 30 mg used for refolding. The chromatogram of the refolded TOSV 6His-NP shows a homogeneous protein with peaks at 97 kDa (3 molecules of TOSV 6His-NP) and 33 kDa (1 TOSV 6His-NP). The NP eluting at 97 kDa corresponds to 63% of the injected protein (4 mg). The two protein pools corresponding to the peaks 97 and 33 kDa were separately used for crystallogenesis trials.

### Protein crystallization

**Figure 8:**
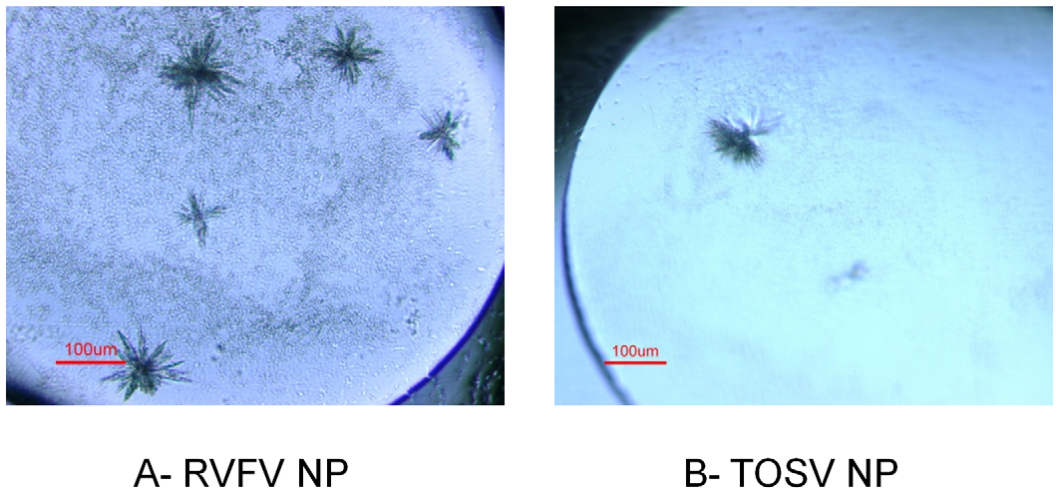
Crystal hits of RVFV and TOSV NP from the commercial kits. Panel A: RVFV NP in 30% PEG 4000, 0.2 M imidazole malate pH 6 (from Stura Footprint Screen, Molecular Dimensions). Panel B: TOSV NP in 33% PEG 600 0.2 M imidazole malate pH 5.5 (from Stura Footprint Screen, Molecular Dimensions).

RVFV NP and TOSV NPs obtained from the non-denaturing production pipeline after TRX-6His removal, as well as the two pools (97 kDa and 33 kDa) of the refolded 6His-TOSV NP were finally concentrated in the SEC buffer up to 6.6 mg/ml, 6.3 mg/ml, 7.4 mg/ml and 3 mg/ml respectively. From the commercial kits, several crystal hits were obtained for RVFV NP (figure 8, panel A), and one condition lead to sea urchin crystals of the 97 kDa MW TOSV NP (figure 8, panel B).

## Discussion

The initial objective of this study was to produce and purify *phlebovirus* NPs for structural studies. According to the previously reported studies, NPs were described as insoluble [23] or HMW complexes [22]. Oppositely to the latter strategy whose goal was to identify the viral organization of Rift Valley fever virus at the macromolecular level, our aim was to find a procedure to produce soluble and LMW NPs. Therefore, we decided to adopt a naive approach testing in parallel several methods and evaluate them on the basis of protein solubility and production of LMW NPs. With these two evaluation criteria, we devised an easy “Go/No Go” pipeline (figure 2) that enables the comparison of the methods and the selection of the best one, in order to reach the required amount of homogeneous NP for crystallogenesis. For several reasons, it was decided to take advantage of the natural diversity within the viral genus. Firstly, screening several homologues can be considered as an additional experimental variable. It has already been demonstrated that it can improve the success rate to find at least one sequence suitable for structural studies [36]. Secondly, the cumulative data of several proteins could benchmark the protocol described in figure 2. Finally, it could be of interest to see if phlebovirus NPs, share preliminary structural features such as oligomerization. Three phleboviruses belonging to distinct phylogenic clusters based on NP sequences [37] were therefore selected, the pairwise sequence identities of the NPs being 50% id. between RVFV and TOSV, 47% id. between RVFV and SFSV and 40% id. between TOSV and SFSV.

Among these three NPs, SFSV NP was the most reluctant to all strategies. Either the protein with a 6His tag is poorly soluble, or it cannot be refolded easily. When expressed in fusion with the TRX-6His sequence, the solubility issue is overcome but the fusion protein was almost refractory to TEV protease cleavage. Moreover, the cleaved and uncleaved forms co-eluted together on IMAC with no rational on the presence/absence of the 6His sequence. Altogether, these data suggest that even if the TRX-6His solubilizes the SFSV NP, the fusion protein organizes in soluble aggregates, as already observed with MBP when it is fused to the human papillomavirus E6 protein [38]. By contrast, the two RVFV and TOSV were soluble and easier to handle. However, sequence analysis for solubility in *E. coli* [39] did not allow predicting of the untracktability of SFSV compared to the two other NPs. Again, it suggests that the success rate of producing a class of proteins depends on the number of homologues tested experimentally. Alternatively, specific point mutations of poorly conserved amino acids between SFSV and the two others NPS might constitute a successful strategy to obtain soluble or stable SFSV NP. Such a study might help to point out critical residues like prolines or cysteines that can be involved in recombinant protein folding/misfolding.

TOSV NP was soluble in both 6His and TRX-6His constructs but the latter tag improved solubility about 20-fold. The tag was cleaved and the SEC chromatogram revealed two NP populations that could correspond to hexameric and trimeric complexes (figure 5, panel B). Surprisingly, the oligomerization states shifted to putative trimers and monomers (panel C) from the purification under denaturing conditions followed by refolding. A much more striking difference was observed for the RVFV NP. Both constructs (6His-NP and NP) from the non-denaturing pipeline eluted mainly as putative trimers (figure 5, panels A and B), whereas 6His-NP after refolding formed putative monomers, dimers and tetramers (panel C). This “2X” multimerization is in agreement with results obtained for refolded RVFV NP that lead to the structure determination of a NP dimer [26] but it differs from the “3X” multimerization of native and refolded TOSV NP and native RVFV NP. Our data suggest that the choice of the purification procedure (*i.e.,* denaturing *vs* non-denaturing conditions) has an impact on the oligomerization state going through two divergent directions and at this stage, we were not able to claim if the difference was due to a refolding artifact or an equilibrium between the two forms.

In a previous study, it was demonstrated that the RVFV NP can dimerize through an interaction involving the N-terminal domain [20]. When cross linking RVFV NP with glutaraldehyde, we did confirm that the protein expressed in *E. coli* is able to dimerize but also to produce tri- and tetramers (figure 6). These latter oligomers almost disappeared in the HMW NP (>300 kDa), suggesting that HMW could behave like RNPs purified from infected Vero cells [20]. For the lower molecular weight complexes, the cross linking with glutaraldehyde resulted in the formation of a ladder that is probably due to a non specific reaction. Since SEC chromatograms of the soluble 6His-RVFV NP and RVFV-NP are almost superimposable, the 6His tag was not responsible for the oligomerization differences.

Two NP pools crystallized (figure 8), one from the non-denaturing pathway (RVFV NP) and one from the refolding one (TOSV NP). In both cases, crystals were obtained with trimeric NPs. These results raise the importance to investigate the production under not only the native conditions but also the denaturing conditions for at least two raisons. Firstly, in contrast with to the soluble fraction of RVFV 6His-NP, refolded 6His-NP remained not degraded, even after refolding. It can be noticed that the degradation of the 6His-NP, when processed in non-denaturing conditions, occurred at an early stage of the production (figure 3), probably during the culture growth or bacterial lysis, whereas the NPs produced as IB are prevented from bacterial proteolysis. Secondly, the putative trimer of TOSV NP that crystallized was obtained in higher amounts after refolding than in the soluble fraction that majorly lead to a heterogeneous oligomer preparation. Nevertheless, refolding from solubilized inclusion bodies met some limits in the purification of NP. When SFSV NP is not soluble and homogeneous even with the TRX tag, the refolding strategy also failed. Moreover, the renatured RVFV NP produced multimers that are different from RVFV and TOSV NPs purified under native conditions, in agreement with a previous study [26]. Although it remains unclear if the differences in the oligomerization states highlight a difference in protein folding, the structure of a phlebovirus NP as a trimer would certainly help understanding the oligomerization determinants of phlebovirus NPs.

### Conclusion

The standard pipeline investigating conditions under both denaturing and non-denaturing conditions was proven to be efficient and could be applied to any recombinant protein. With no need of further refinement, two out of the three phlebovirus NPs were produced and purified in suitable amount and quality for crystallogenesis. Trimeric forms of RVFV and TOSV NPs yielded crystals. This result is a starting point for structural studies aiming at the elucidation of the RNA encapsidation mechanism, a targetable step for antiviral research [40].

## Supporting Information

Abstract S1 Translation of the abstract into French by Bruno Coutard.
(0.03 MB DOC)
