# Supplementary material for: Comparative Production Analysis of Three Phlebovirus Nucleoproteins under Denaturing or Non-Denaturing Conditions for Crystallographic Studies
Source: PLoS Negl Trop Dis. 2011 Jan 4;5(1):e936. doi: 10.1371/journal.pntd.0000936 (PMC3014985; doi:10.1371/journal.pntd.0000936)
Supplement: Abstract S1 — Translation of the abstract into French by Bruno Coutard. (0.03 MB DOC) [file pntd.0000936.s001.doc]

Translation of the abstract into French by Bruno Coutard

Les nucleoprotéines (NPs) forment une capside autour du génome ARN de polarité négative des Phlébovirus. Produites sous forme recombinantes, ces NPs s’organisent en oligomères hétérogènes, empêchant la caractérisation du mécanisme d’encapsidation par les études cristallographiques. Afin de résoudre ce problème d’hétérogénéité, nous avons mis au point un protocole dans lequel les productions en conditions native ou par dénaturation/renaturation sont testées et comparées. Cette procédure a été évaluée avec trois nucléoprotéines de Phlébovirus, permettant ainsi de définir les conditions de production optimales pour chacune d’entre elles. La NP du virus de la vallée du Rift a pu être purifiée en condition native sous forme de trimère qui a conduit à la production de cristaux, alors qu’après dénaturation et renaturation des corps d’inclusion, la protéine s’organise en dimère. La NP du virus Toscana se produit, elle, sous forme de trimère en conditions native ou après renaturation ; cette dernière condition permettant de produire suffisamment de protéine pour initier les tests de cristallisation. En revanche, la production de la NP du virus de la fièvre à phlébotome, souche sicilienne a échouée dans toutes les conditions expérimentales. Cette étude comparative devrait permettre de choisir de manière rationnelle entre les conditions native ou par dénaturation/renaturation, le mode de production conduisant à l’état d’oligomérisation approprié. La structure du monomère de la NP du virus de la vallée du Rift a été récemment résolue à partir d’une forme renaturée, et nous pensons que la procédure que nous avons développée permettra d’apporter des informations structurales complémentaires sur l’oligomérisation et l’encapsidation, une étape clé dans le cycle viral.
